# Initial Characterization of the FlgE Hook High Molecular Weight Complex of *Borrelia burgdorferi*

**DOI:** 10.1371/journal.pone.0098338

**Published:** 2014-05-23

**Authors:** Kelly A. Miller, Md. A. Motaleb, Jun Liu, Bo Hu, Melissa J. Caimano, Michael R. Miller, Nyles W. Charon

**Affiliations:** 1 Department of Microbiology, Immunology, and Cell Biology, Robert C. Byrd Health Sciences Center, West Virginia University, Morgantown, West Virginia, United States of America; 2 Department of Microbiology and Immunology, East Carolina University, Greenville, North Carolina, United States of America; 3 Department of Pathology and Laboratory Medicine, Medical School, University of Texas Health Science Center at Houston, Houston, Texas, United States of America; 4 Department of Medicine, University of Connecticut Health Center, Farmington, Connecticut, United States of America; 5 Department of Biochemistry, Robert C. Byrd Health Sciences Center, West Virginia University, Morgantown, West Virginia, United States of America; University of Toledo School of Medicine, United States of America

## Abstract

The spirochete periplasmic flagellum has many unique attributes. One unusual characteristic is the flagellar hook. This structure serves as a universal joint coupling rotation of the membrane-bound motor to the flagellar filament. The hook is comprised of about 120 FlgE monomers, and in most bacteria these structures readily dissociate to monomers (∼ 50 kDa) when treated with heat and detergent. However, in spirochetes the FlgE monomers form a large mass of over 250 kDa [referred to as a high molecular weight complex (HMWC)] that is stable to these and other denaturing conditions. In this communication, we examined specific aspects with respect to the formation and structure of this complex. We found that the Lyme disease spirochete *Borrelia burgdorferi* synthesized the HMWC throughout the *in vitro* growth cycle, and also *in vivo* when implanted in dialysis membrane chambers in rats. The HMWC was stable to formic acid, which supports the concept that the stability of the HMWC is dependent on covalent cross-linking of individual FlgE subunits. Mass spectrometry analysis of the HMWC from both wild type periplasmic flagella and polyhooks from a newly constructed Δ*fliK* mutant indicated that other proteins besides FlgE were not covalently joined to the complex, and that FlgE was the sole component of the complex. In addition, mass spectrometry analysis also indicated that the HMWC was composed of a polymer of the FlgE protein with both the N- and C-terminal regions remaining intact. These initial studies set the stage for a detailed characterization of the HMWC. Covalent cross-linking of FlgE with the accompanying formation of the HMWC we propose strengthens the hook structure for optimal spirochete motility.

## Introduction

Spirochetes are a ubiquitous monophyletic motile phylum of bacteria, with many species causing disease [1]. These organisms, which are either helical or flat-waves, have a unique structure, as the organelles responsible for motility, referred to as the periplasmic flagella (PFs), reside between the outer membrane and protoplasmic cell cylinder. Each PF is attached to one cell end and extends towards the opposite end of the cell. Rotation of these relatively rigid organelles generates perturbations and wave propagation along the length of the relatively flexible cell cylinder such that the spirochetes efficiently translate and reverse directions even in viscous gel-like material and *in vivo*. Thus, the PFs need to exert substantial force on the cell cylinder to generate these backward moving waves [2]–[8].

Spirochetes also have several unique attributes with respect to their PFs. Each PF consists of a membrane imbedded motor where the basal body complex resides, a hook region that serves as universal joint, and the filament [2], [3]. Thus, in many respects, the PF is similar in structure to the external flagellum of other bacteria. In contrast to the flagellum filament of most other bacteria, the PF filament consists of multiple protein species with a protein sheath surrounding the entire or part of the filament core. For example, *Treponema* and *Brachyspira* species have three different filament core proteins referred to as FlaB1, FlaB2, and FlaB3, and a sheath protein referred to as FlaA [2], [3]. *Borrelia burgdorferi* has only one core protein referred to as FlaB and a sheath protein referred to as FlaA. Mutants that lack any of these proteins are deficient in motility [9], [10]. In *B. burgdorferi*, mutants that lack FlaB are completely deficient in PFs, are non-motile, and are non-infectious [11], [12]. The PF motor is also unique. Recent cryoelectron microscopy (cryo-EM) results suggest a specialized collar is associated with the motor in the vicinity of the peptidoglycan layer, but the function of the collar is presently unknown [13], [14]. In addition, its cytoplasmic C-ring complex is considerably larger than that of other bacteria. The larger C-ring complex in spirochetes is believed to enable the PFs to exert sufficient torque such that the PFs promote wave propagation along the cell length [14].

Another unique attribute of spirochetes, which is the focus of this report, is their flagellar hook. The hook is a hollow tubular structure, approximately 61 nm long in *B. burgdorferi*, and is composed of at least 120 FlgE proteins [15]. FlgE is a well conserved protein among bacteria. Several reports indicate that FlgE of spirochetes form a stable, high molecular weight complex (HMWC) that is unique. Thus, western blots of whole cell lysates and in some cases purified PFs of *B. burgdorferi*, *Treponema denticola*, *Treponema pallidum*, and *Treponema phagedenis* probed with polyclonal FlgE antibodies react with a protein(s) located near the top of a gel when analyzed by sodium dodecyl sulfate polyacrylamide gel electrophoresis (SDS-PAGE). Sometimes the HMWC resides even in the stacking gel, with a mass greater than 250 kDa [15]–[18]. This HMWC is considerably larger than the monomer (∼50 kDa) [15]–[19]. In contrast, in bacteria with external flagella, western blots probed with FlgE antibody show reactivity exclusively at the molecular weight of monomeric FlgE [20], [21]. The ability of spirochete FlgE to form a HMWC is similar to electrophoretic patterns found with covalent cross-linked proteins. For example, the head proteins of phage HK97 that infects *Escherichia coli,* and the pili proteins of Gram positive bacteria such as *Streptococcus pyogenes* and *Corynebacterium diptheriae* also form HMWCs [22], [23]. In addition, HMWCs from spirochete FlgE, HK97, and Gram positive pili are stable to a variety of harsh treatments and chemical agents, including boiling, guanidine hydrochloride, and 8M urea [15], [18], [22], [23]. Accordingly, we and others hypothesize that the FlgE HMWC of *B. burgdorferi* and other spirochetes is attributed to covalent cross-linking among its individual proteins, which may be essential for optimum motility and virulence [15], [18].

The characterization of the spirochete FlgE HMWC has been limited. Because FlgE is normally in small amounts (less than 9 µg per liter of cells of *B. burgdorferi*), chemical characterization is difficult. Several basic questions arise concerning these complexes. Are the HMWCs formed at all phases of *B. burgdorferi in vitro* growth, and are they formed *in vivo* in an infected animal? Are HMWCs stable to formic acid as are the head proteins of HK97 and pili of Gram positive bacteria [22], [23]? Is there more than one protein, such as another basal body protein being part of the HMWC? Does the HMWC consist of a polymer of the complete FlgE protein, or is there truncation of either of its ends? In this communication, using the Lyme disease spirochete *B. burgdorferi* as a model that forms the FlgE HMWC, we answer the above questions.

## Materials and Methods

### Ethics statement

All animal experimentation was conducted following the Guide for the Care and Use of Laboratory Animals and in accordance with protocols reviewed and approved by the University of Connecticut Health Center Institutional Animal Care and Use Committee.

### Bacterial strains and growth conditions

High passage, avirulent *B. burgdorferi* strain B31A [24], and low passage virulent strain B31A3 [25], were grown at 33°C (except as noted) in the presence of 3% carbon dioxide in BSKII growth medium [11]. Cell counts were determined using flow cytometry as previously described [26]. These strains were originally obtained from P. Rosa, Rocky Mountain Laboratories, MT. The virulent, low passage strain B31MI [27] was grown *in vitro* at 23°C, 37°C, and in *in vivo* in dialysis membrane chambers (DMCs) in Sprague-Dawley rats [28]. For *in vivo* growth, sterilized dialysis membranes with a molecular weight cut-off of 8000 Da were filled with ∼ 9 ml BSKII medium containing late logarithmic phase *B. burgdorferi* strain B31MI diluted to a starting density of 3000 spirochetes/ml. DMCs were aseptically implanted into the peritoneal cavities of female Sprague Dawley rats, and then harvested 14 days post-implantation as previously described [28]. *B. burgdorferi* recombinant FlgE (rFlgE) was expressed in *E. coli* BL21 cells grown at 37°C in Luria-Bertani broth containing 100 µg/ml ampicillin and purified as previously described [15].

### Construction of the Δ*fliK1* mutant

The *fliK* mutant, Δ*fliK1*, was derived from high passage *B. burgdorferi* strain B31A; it was constructed as follows: The *fliK* gene (gene locus *bb0285*; 1179 base pairs) is located in the large *flgB* operon consisting of 26 genes [27], [29]. This gene overlaps with the upstream *flgD* gene by 14 base pairs and is separated from the downstream *bb0286* gene by eight base pairs. *fliK* was inactivated by replacing its coding sequence using overlapping PCR with an *aadA* streptomycin/spectinomycin resistance coding cassette that results in non-polar insertions [30]. Specifically, PCR was used to amplify three regions. First, each DNA region was amplified separately using primer pairs P1–P2 (5′-flanking DNA, *flgD*-*flgE*), P3–P4 (*aadA* coding sequence), and P5–P6 (3′-flanking DNA, *bb0286*-*bb0287*, Table 1). Second, a PCR product was obtained using primers P1-P4 with the purified DNA products *flgD*-*flgE* and *aadA* as templates. Third, the desired PCR product was obtained using primers P1–P6 with the purified DNA products *flgD*-*flgE-aadA* and *bb0286*-*bb0287* serving as templates. The final 2836 *flgD*-*flgE-aadA*-*bb0286*-*bb0287* base pair PCR product was gel purified and cloned into the pGEM-T Easy vector (Promega Inc.) to yield the *fliK:aadA*-pGEM plasmid. The integrity of the *fliK* inactivation plasmid was confirmed by PCR and restriction mapping. A PCR-amplified linear DNA product containing the *fliK* inactivation region was electroporated into B31A competent cells [24]. The electroporated cells were spread on semisolid BSKII medium containing 80 µg/ml streptomycin. Resistant clones were picked and analyzed by PCR using primers P1–P6 to confirm deletion of *fliK*. One clone, Δ*fliK1*, was found to have the desired mutation and further characterized.

**Table 1:** Primers used to generate the Δ*fliK* mutant*.

Primer #	Name	Direction	Sequence (5′–3′)
1	FliK KO F	Forward	agattacaagcaaaagtaac
2	FliK-Str-KO-Part 2-F	Reverse	ggtagtcggcaaataagaattttcttacgatgtagata
3	FliK-Strep-R	Forward	catcgtaagaaaattcttatttgccgactaccttggtg
4	FliK-Str-F	Reverse	tggagtagtgtgtatatgagggaagcggtgatcgccga
5	FliK-Str-KO-Part 1-R	Forward	caccgcttccctcatatacacactactccaatgaact
6	FliK KO R2	Reverse	tagaacctactttcgaaagctaagcg

*Underlined sequences indicate overlapping base pairs.

### SDS-PAGE and western blot analysis

SDS-PAGE and immuoblotting were conducted using standard techniques [15], [31]. Molecular weight markers were obtained from Bio-Rad (cat no. 161–0376). All samples except as noted were boiled in sample buffer for at least 5 min. Samples were electrophoresed in 8% polyacrylamide gels, and silver stained with Protea Biosciences, Inc. silver staining kit or with Bio-rad Sypro protein gel stain. For western blotting, 1 µg total protein (unless noted otherwise), as determined by the Bradford protein assay (Bio-Rad), was subjected to SDS-PAGE and transferred onto polyvinylidene difluoride (PVDF) membranes. Immunoblots were probed with rabbit polyclonal antibody directed against *B. burgdorferi* FlgE [15], and secondary horse radish peroxidase (HRP)-labeled polyclonal donkey anti-rabbit antibodies obtained from GE Healthcare. FlaB was detected using monoclonal antibody H9724 to *B. burgdorferi* FlaB as previously described [11], [32]. An enhanced chemiluminescent detection system was used to assay for reactivity (Pierce). Relative amounts of FlgE were determined by densitometry. After film development, films were scanned, and relative band intensities were determined using Image Quant software (GE Healthcare Life Sciences).

### PFs and polyhook isolation

The PF isolation method of Sal *et al*. [15] was modified to increase the yield of the FlgE HMWC and to purify polyhooks. One liter of late logarithmic phase wild-type or Δ*fliK1* mutant cells at a density of 1.5×10^8^ cells/ml were divided into four 250 ml portions. Cells from each portion were harvested by centrifugation (8,000×g, 20 min, 4°C), and the cell pellet was suspended in 28 ml of 150 mM phosphate buffered saline, pH 7.4 (PBS) and centrifuged (8,000×g, 15 min, 4°C). After suspending each pellet in 30 ml 0.15 M Tris, pH 6.8, (T buffer) the cells were centrifuged as indicated above, then resuspended in 15 ml of T buffer. The cell suspension was stirred for 10 min (23°C), and Triton X-100 was added (20% stock in sterile water) to a 2% final concentration. The cell suspension was stirred for 30–60 min at 23°C, or until the outer membranes were disrupted as observed by darkfield microscopy. Such spirochetes appeared thinner after treatment. Darkfield microscopy was carried out as previously described [33]. Mutanolysin (Sigma cat no M9901-50KU) was slowly added from a 200 µg/ml stock in H_2_0 to 20 µg/ml final concentration and stirred (2 hr., 23°C, followed by overnight, 4°C). Cells were completely lysed at this stage as revealed by darkfield microscopy. MgSO_4_ was added to a final concentration of 1.7 mM followed by stirring for 10 min at 23°C. The suspension was centrifuged at 17,000×g for 15 min at 4°C to remove cell debris. The supernatant was carefully removed and 20% polyethylene glycol (PEG 8000) in 1 M NaCl was added to 2% final concentration and thoroughly mixed. After incubation on ice for 30 min, the solution was centrifuged (27,000 xg, 30 min, 4°C), and the supernatant fluid was discarded. The PFs or polyhooks were resuspended in 0.1 M KCl, 0.5 M sucrose, 0.1% Triton X-100, 50 mM sodium bicarbonate, pH 11 for 1 hour at 23°C [34], and the final products were recovered by centrifugation at 80,000×g, 45 min, 4°C. The isolated PFs or polyhooks were pooled, resuspended in a small volume of water (∼1 ml), and stored at 4°C with 0.2% sodium azide.

### Electron microscopy

Cryo-electron tomography (Cryo-ET) was used to localize polyhooks within cells [13], and cryo-electron microscopy (cryo-EM) was used to test for the integrity of isolated PFs and polyhooks. Briefly, bacterial cultures were mixed with 15 nm gold clusters, which were used as fiducial markers. EM grids containing samples were frozen rapidly and stored in liquid ethane. Two dimension images and tilt series were collected at 300 kV using a Polara G2 electron microscope (FEI company) connected to a 16 megapixel charged coupled device camera (Tvips GmbH, Germany). The tilt series images were aligned and reconstructed using IMOD software package [35]. In total, 9 cryo-tomograms were reconstructed from the *ΔfliK* mutant. One representative reconstruction was segmented using three-dimensional (3-D) modeling software Amira (Visage Imaging). 3-D segmentation of the flagella, outer membrane and cytoplasmic membrane was manually constructed. The surface model from the averaged flagellar motor [13] was computationally mapped back into the original cellular context. In nine different cells, the length of 50 polyhooks was measured; this data is depicted in a bar graph and the average length of the 50 polyhooks was determined.

### Mass spectrometry (MS) analysis

For mass spectrometry analysis (Protea Biosciences, Inc.), isolated PFs or polyhook samples were subjected to SDS-PAGE, and the gels were silver- or Sypro-stained, followed by in-gel digestion with trypsin. Gel bands of interest were excised, washed with 150 mM ammonium bicarbonate and acetonitrile, and lyophilized. The dried gel bands were treated with dithiothreitol and iodoacetamide followed by digestion with 12.5 ng/μl trypsin. The digested peptides were analyzed by LC-ESI MS using a QTrap5500 (AB Sciex Toronto, Canada). Peptides were separated at 35°C on a Kinetex 100×2.1 mm C_18_ column on a Shimadzu LC-20AD HPLC (Tokyo, Japan) using a 120-minute gradient. The mass range acquired (m/z) was 100-1000, and the three most intense multiply charged ions with ion intensities above 50,000 in each MS scan were subjected to MS/MS. The MS/MS data were searched for matches to protein databases using ABI Protein Pilot software 3.0. Only those peptides that were identified with >95% confidence were considered.

## Results and Discussion

### FlgE HMWC formation during different phases of growth

**Figure 1:**
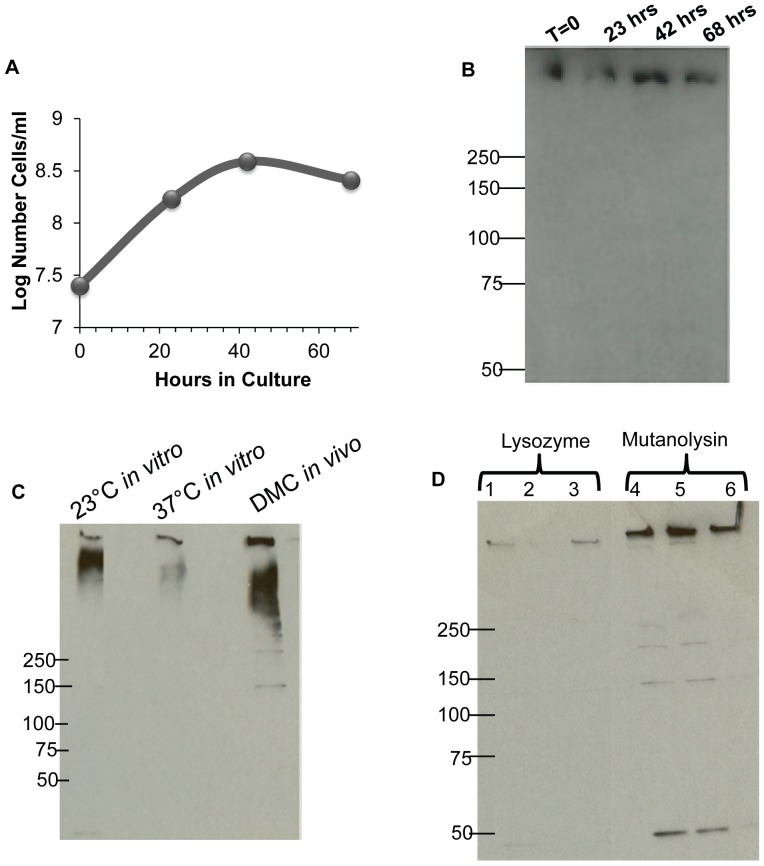
Synthesis of the HMWC under different growth conditions, and its isolation using mutanolysin. (A) Growth curve of *B. burgdorferi* B31A and (B) accompanying immunoblots of whole cell lysates probed with anti-FlgE, with samples taken at designated time points. [The position of molecular weight markers is indicated in Figures 1B–1D (50 – 250 kDa). (C) Immunoblots probed with anti-FlgE of strain B31MI grown at designated temperatures *in vitro*, and *in vivo* in DMC in rats. The amount of protein loaded was normalized to FlaB [32]. (D) Immunoblots probed with anti-FlgE of three independent PF isolation preparations from strain B31A using either the lysozyme or mutanolysin procedure. Approximately 0.5 µg of protein were loaded in each lane.

We first tested if the HMWC was synthesized at all phases of growth. Conceivably, cross-linking could occur in a specific growth phase analogous to, for example, *Escherichia coli* modifying its preformed unsaturated fatty acids by cyclopropanation primarily in stationary phase [36]. Furthermore, specific σ^S^-dependent genes have been shown to be upregulated in *B. burgdorferi* in stationary phase and during growth under specific conditions [37]–[39]. Accordingly, samples were taken throughout the *in vitro* growth cycle of *B. burgdorferi*, the cells were lysed, and then tested for the HMWC by western blot (Figure 1, A, B). We found that the HMWC was present in all phases of growth, and trace amounts of the monomer were sometimes detected. Often the HMWC localized at the stacking-running gel interface, or as previously noted, within the stacking gel itself [15]. No differences were seen in the HMWC formation throughout the cycle. These results suggest that FlgE HMWC formation is not growth phase dependent.

### FlgE HMWC formation at different temperatures and growth *in vivo*

Both the tick and mammal are the main habitats of *B. burgdorferi* in nature [39]–[41], and protein expression markedly varies in these two hosts. [39], [41]. In addition, many of the genes that are encoded by the 21 circular and linear plasmids present in *B. burgdorferi* are differentially expressed in the tick and mammal [40]. We tested whether HMWC formation occurred under conditions that mimic growth in the tick (23°C) and the mammal (37°C). In these experiments, we used a strain of *B. burgdorferi* which was virulent (B31M1), as such strains, in contrast to avirulent strains, show major differences in protein profiles when cultured under these two temperatures [42]. We found that the B31MI strain grown *in vitro* at 23°C and at 37°C formed the HMWC (Fig. 1C). Similar results were found with cloned virulent strain B31A3 (K. Miller and N. Charon, not shown). These results indicate that the HMWC was synthesized under conditions that mimic growth in the tick and mammal. In addition, the results also indicate that the change in the protein profile that accompanies growth under these two conditions does not influence HMWC formation. We also asked if HMWC formation was a result of *in vitro* growth. *B. burgdorferi* cells grown *in vivo* are known to markedly change their transcriptional and protein profile compared to cells grown *in vitro* [28], [39]–[41]; conceivably, the HMWC may not form in the mammal. Accordingly, we tested if the HMWC is synthesized *in vivo* by implanting B31MI cells in DMC chambers in rats [28]. The cells were harvested at 14 days, centrifuged, and analyzed as before using immunoblotting. We found that the FlgE HMWC was present in cells grown *in vivo* in the DMCs similar to cells grown *in vitro* (Fig. 1C). In sum, the FlgE HMWC was synthesized in *B. burgdorferi* in all *in vitro* growth conditions tested and also *in vivo*. In all conditions tested, the FlgE HMWC did not migrate as a discrete band or size, and it often localized to the stacking gel (Fig 1B, C). Conceivably, the HMWC could be composed of multiple masses that migrate anomalously in SDS PAGE. Protein crosslinking in the pili of *Corynebacterium diphtheriae* also results in diffuse bands upon SDS PAGE [23].

### Optimization of PFs and FlgE isolation

**Figure 2:**
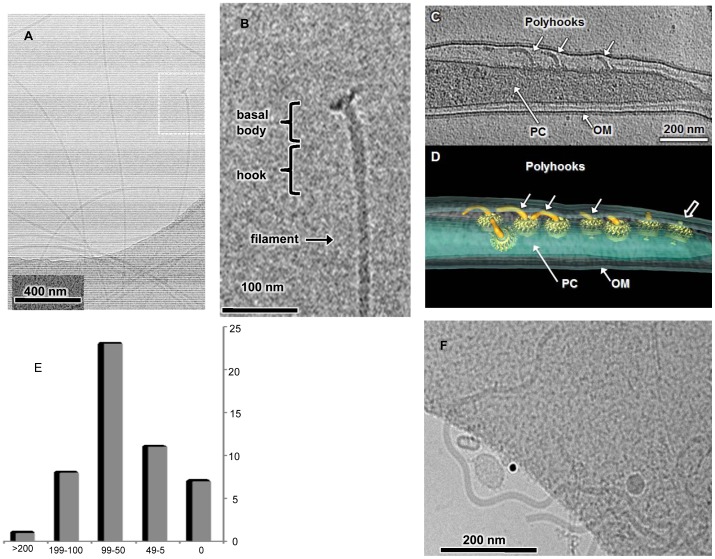
Cryo-EM image of PFs, *ΔfliK* mutant, and polyhooks. (A) PFs isolated from wild-type *B.burgdorferi* B31A. (B) The hook basal body structure that is boxed in A is enlarged in B and accompanying structures identified. (C) The *ΔfliK* mutant with three flagellar polyhooks (∼50 – 150 nm in length) localized in the periplasmic space between the outer membrane (OM) and protoplasmic cell cylinder (PC). (D) A 3D surface rendering of polyhooks and motors, suggesting the polyhooks are variable in length. (E) the lengths of 50 polyhooks in 9 intact *Δflik* mutants was determined (F) Polyhooks isolated from the *ΔfliK* mutant using the mutanolysin procedure.

**Figure 3:**
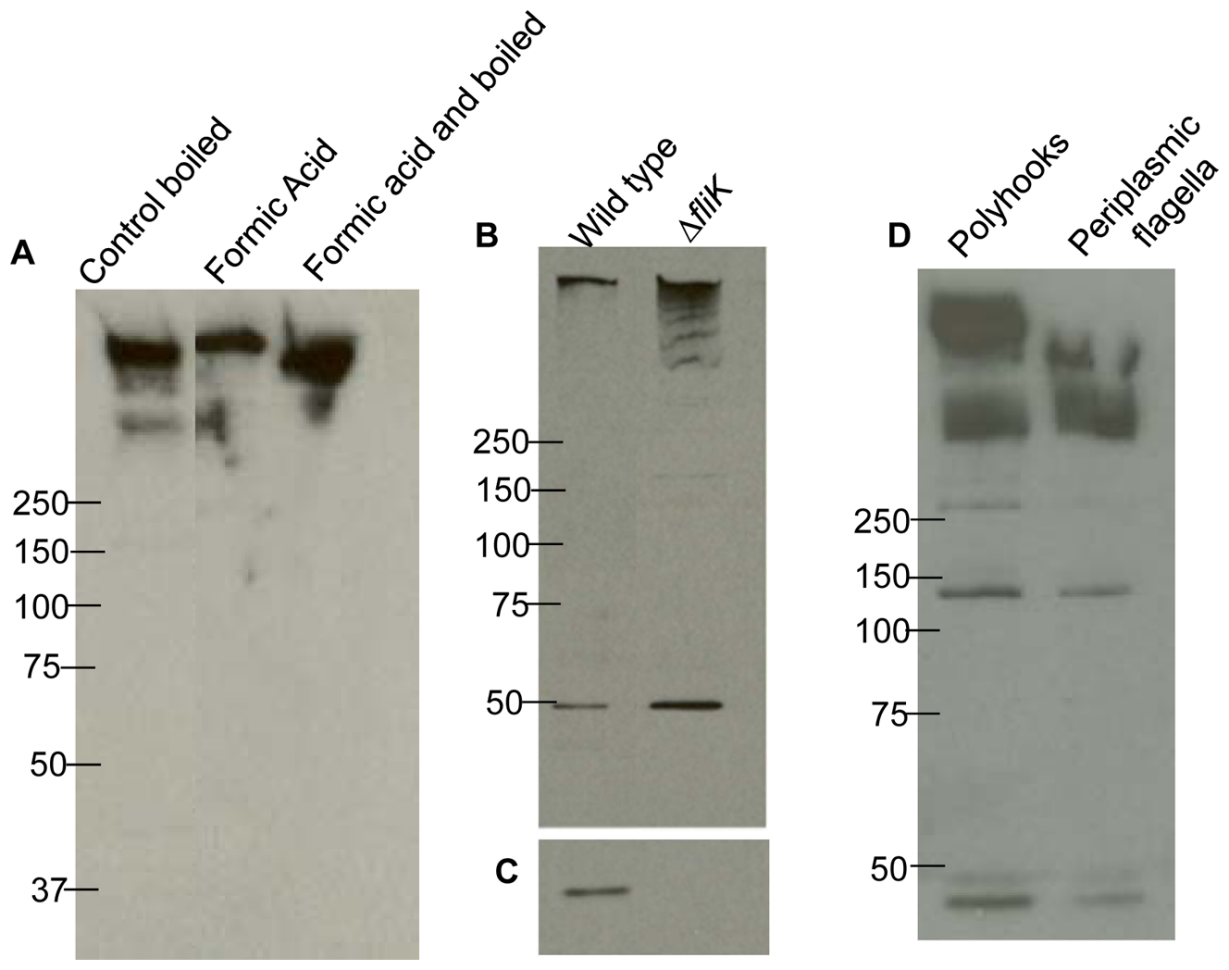
Immunoblot analysis of PFs and Δ*fliK* mutant. (A) Isolated PFs were treated with formic acid for 2 hrs at room temperature (lane 2) or 100°C for 15 minutes (lane 3). After formic acid treatment, sample buffer was added and the gel was loaded without additional boiling. The control was boiled for 15 min. 1 µg protein was loaded per lane, and the blot was probed with FlgE antisera. The position of molecular weight markers is indicated in Figures 3A, 3B and 3D (50 – 250 kDa). (B) Whole cell lysates (1.0 µg) of wild-type and *ΔfliK* mutants probed with FlgE and (C) FlaB antisera. Reactivity was at 42 kDa with anti-FlaB. (D) Isolated polyhooks and PFs probed with FlgE antiserum.

We began characterization of the HMWC. In our initial studies of *B. burgdorferi* FlgE, we used a method of lysing cells with myristate followed by lysozyme to purify PFs [15]. We found that this method was inadequate for chemical analysis of the HMWC (the yield was less than 1 µg, which is estimated to be less than 10% of the total FlgE protein in a liter of cells). Several modifications were made for optimization. These included substituting Triton X-100 for myristate, and mutanolysin for lysozyme. Triton X-100 has been shown to successfully remove the outer membranes of several spirochete species, including *T. phagedenis* [43], *T. pallidum* [44], *T. denticola* [45], and *B. burgdorferi* [46]. Although mutanolysin and lysozyme both hydrolyze the β-N-acetylmuramyl- (1→4)- N -acetylglucosamine linkage of the bacterial cell wall polymer peptidoglycan-polysaccharide, their activities are differentially effected by peptidoglycan modifications. Specifically, N- and O-acetylation inhibit lysozyme activity in a concentration dependent manner; however, mutanolysin maintains its effectiveness in the presence of these modifications [47]–[51]. We found that these changes increased the yield of the HMWC greater than three-fold (Fig. 1D). We also found that isolated PFs were often contaminated with lipid material as seen in cryo-EM. Using an alkaline wash as done for *S. enterica* flagella [34], isolated PFs were free of the lipid containing material (Fig. 2A,B). The total yield of PFs from a liter of cells varied from 2–4 mg. To begin characterization of the HMWC, we purified PFs, and subjected the fraction obtained to formic acid treatment. Stability to formic acid is the hallmark method used to show protein crosslinking of Gram positive pili [23]. As can be seen in figure 3A, boiling for 15 minutes with or without 88% formic acid failed to disrupt the HMWC. Additionally, the FlgE HMWC was observed when the samples were treated with formic acid without boiling, indicating that the HMWC is not an artifact produced by boiling (Fig. 3A). These results support the conclusion that the HMWC is the result of covalent protein-protein cross-linking. We attempted to determine the size of the HMWC. Hooks were prepared from isolated PFs and run on a Superose 12 molecular sieve column with or without pretreatment with 1% SDS. In both cases, the protein eluted as a rather broad band, most of which was in the void volume, indicating the HMWC had a mass at least as large as 1 MDa (not shown).

### Analysis of a *fliK* mutant

The characterization of the spirochete FlgE HMWC has been limited because FlgE is normally in limiting amounts. Therefore, we considered the possibility that polyhooks obtained from *B. burgdorferi* would be a rich source of the HMWC. Although *fliK* mutants in other bacteria including *S. enterica* [52], [53] and *T. denticola* [17] produce large polyhook structures, we did not know if a similar mutant in *B. burgdorferi* would also produce polyhooks. In addition, we were curious if the polyhooks that are synthesized by the mutant would form FlgE HMWCs. Accordingly, we constructed a non-polar *ΔfliK1* mutant of *B. burgdorferi* by allelic exchange mutagenesis. The *ΔfliK1* mutant was non-motile, and cryo-EM indicated that polyhooks structures were produced and localized to the periplasmic space (Fig. 2 C,D; Movie S1); furthermore, flagellar filaments did not appear attached to the polyhooks (Fig. 2 C, D). The length of 50 polyhooks in 9 intact *Δflik* mutants was determined to range from 0 (no hooks attached to motors) to 846 nm, with a mean of 78 nm (Fig 2E). These results suggest that the *ΔfliK* mutant of *B. burgdorferi* is similar to those found in other bacteria, except in this case, the polyhooks are located in the periplasmic space rather than externally and exposed to the medium.

The *ΔfliK* mutant was further analyzed. Western blot analysis of whole cell lysates indicated that most of the reactivity of both the wild-type and the *ΔfliK* mutant formed a HMWC near the top of the gel, greater than 250 kDa (Fig 3B). A previous report demonstrated that cell lysates of *flgE* mutant cells failed to react with the FlgE polyclonal antiserum [15]. Small amounts of the monomer were detected in the mutant, as with the wild-type. Based on densitometry analysis, we estimate that the Δ*fliK1* mutant produces 4–5 times the HMWC as that of the wild-type. We also tested if the Δ*fliK1* mutant produced flagellin by probing cell lysates with FlaB antiserum. In other species of bacteria, *fliK* mutants vary in their ability to continue to synthesize flagellin [54]–[57]. Although strong reactivity was noted with the wild-type, no reactivity was noted in the mutant (Fig. 3C), consistent with cryo-EM images (Fig. 2C, D). These results are similar to those of *S. enterica fliK* mutant cells, as such mutants fail to produce flagella filaments [58], [59]. We purified the polyhooks from the Δ*fliK1* mutant for further immunoblot and cryo-EM analysis. Similar to results with whole cell lysates, FlgE antibody reactivity remained at the HMWC, with some reactivity with the monomer and at approximately 270 and 150 kDa (Fig. 3D). This pattern was similar to that seen with isolated wild-type PFs. Cryo-EM of this fraction identified large polyhook structures similar to those found in other bacteria [17], [59] (Fig. 2F) with a diameter of 16 nm. However, these isolated polyhooks were longer (>200 nm, n = 15) than the mean of 78 nm observed in the periplasmic space of intact cells (Figure 2C,D,E). Therefore, our isolation procedure likely enriches for relatively long polyhooks. The Δ*fliK1* mutant yielded 1–2 mg protein per liter in the isolated polyhook fraction, indicating that it would be an excellent source of the HMWC.

Finding polyhooks produced by this mutant is intriguing for several reasons. In bacteria, the length of the hook is tightly controlled. In the model system of *S. enterica*, interactions of FliK with FlhB determine hook length [52], [54], [59]–[62]. FliK acts as a molecular ruler that is secreted into the medium, and when the hook reaches proper length, FlhB undergoes a conformational change, and FlgE ceases to be added to the end of the forming hook. The cell then begins σ^28^-initiated transcription, and synthesis of the filament protein FliC then commences with concomitant secretion to the growing flagellum [62]. *B. burgdorferi* does have a FlhB homolog (*bb0272*), so it is likely that a similar mechanism exists. However, because the PFs reside in the periplasmic space, if the *S. enterica* model applies to *B. burgdorferi*, FliK would be excreted into this space rather than into the medium as occurs in *S. enterica*. Furthermore, *B. burgdorferi* is unique, as it lacks the transcription cascade control of flagella gene regulation [2], [3], [15], [63], [64]. It lacks, for example, both the σ^28^ transcription factor and the corresponding promoter recognition sequences [2], [3], [27]. Several lines of evidence indicate that *B. burgdorferi* relies instead on translational control for flagella synthesis [15], [63], [64]. Conceivably, perhaps there is a translational control switch that allows *flaB* to be translated once the proper hook length is formed. In support of this notion, because the Δ*fliK1* mutant is unable to stop synthesis of the hook once it reaches its proper length, we find that the resulting mutant also lacks FlaB (Fig. 3C).

### MS of the HMWC

**Figure 4:**
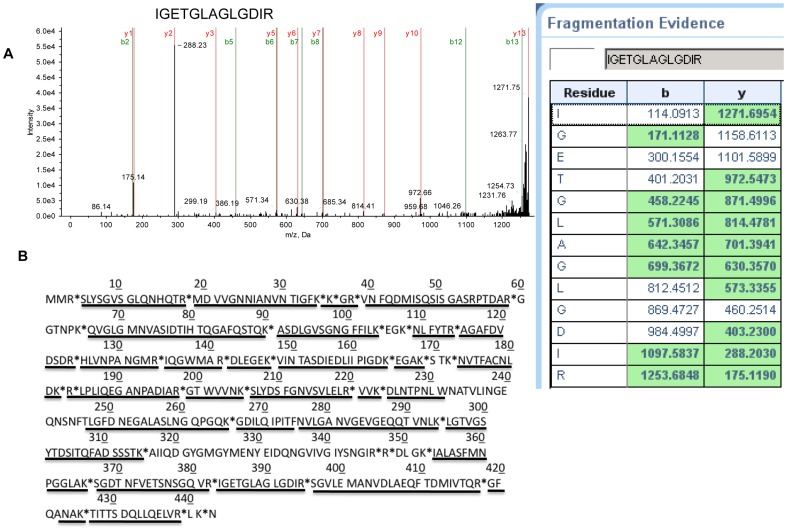
MS analysis of the HMWCs isolated from PFs and polyhooks. The HMWCs were excised, trypsin-digested and analyzed by MS. (A) MS/MS peaks for one of the identified peptides and accompanying fragmentation evidence (aa 383–395). (B) Peptides identified on the FlgE sequence obtained by MS analysis of the HMWC are underlined. * indicate trypsin cut sites.

The revised PF and polyhook purification procedure allowed us to obtain sufficient material for MS analysis of the HMWC. We wanted to determine if other proteins were associated with this complex. We also wanted to determine if formation of the crosslinked HMWC resulted in truncation of either end of FlgE protein, analogous to the sortase-catalyzed reaction in gram-positive pili [65]. The protein composition of the HMWCs obtained from PFs and polyhooks was assessed directly by in-gel trypsin digestion followed by MS analysis (Fig. 4). The MS pattern of one of the peptides is illustrated (Fig. 4A). In 17 MS analyses of the HMWC, no other proteins were consistently identified in the HMWCs, and in many experiments, FlgE was the sole protein. For example, in one experiment with the HMWC obtained from PFs, 27 different peptides were identified, and all were derived from FlgE (Table S1). Similarly, in an experiment with the polyhook, 14 different peptides were identified, and again all were FlgE derived (Table S2). These results indicate that FlgE is the sole protein of the HMWC. We also asked if the N- and C-terminal regions were present in the HMWC. As can be seen from Fig 4B, 85% of the FlgE sequence was present in the complex, including peptides at the N- and C-terminal regions. These results suggest that the HMWC is not a cross-linked product of truncated FlgE. Now that we can obtain sufficient material for MS analysis from both wild-type cells and from a *ΔfliK* mutant, we are actively determining which amino acids form the cross-link, and the precise mechanism of this cross-linking.

### Why would cross-linking occur in the flagellar hooks of spirochetes, but not in the hooks of bacteria with external flagella?

Cross-linking of proteins in bacteria is rare, and when present it results in strengthening protein-protein interactions [66]. The hook acts as a universal joint, transferring the rotational motion of flagellar motors to filaments. In addition, this region has recently been shown to be the most flexible part of the flagellar apparatus; it even buckles when externally flagellated bacteria reverse direction of rotation [67]. Moreover, *Vibrio alginolyticus* exploits this buckling for optimal cell reorientation during swimming [67]. In spirochetes, in contrast to bacteria with external flagella, not only do cells often reverse direction, the rigid PFs exert force on the cell cylinder to generate backward moving waves for translation [2], [4]–[8]. We hypothesize that FlgE cross-linking is essential for the spirochetes to avoid flagellar buckling and allow the cell to reverse directions. This cross-linking also allows the hook to be sufficiently strong such that the PFs can exert adequate force against the cell cylinder to generate the waves responsible for motility. Our results suggest that *B. burgdorferi* FlgE crosslinking occurs in the periplasm during or after the FlgE proteins assemble to form the hook [15]. Whether this crosslinking is self-catalyzed or catalyzed by another protein is not yet known. Searching the *B. burgdorferi* genome did not identify any gene coding a presumptive transglutaminase or sortase protein, nor does the FlgE sequence contain the sortase motif [23].

## Supporting Information

Movie S1 **3D visualization of the ΔfliK mutant**. A 3D reconstruction was generated by cryo-electron tomography of frozen hydrated bacterial culture of the ΔfliK mutant. The movie first shows the slices through the 3D reconstruction, then 3D surface rendering of major features, such as the outer membrane, the inner membrane, and the flagellar motors. In particular, the movie highlights the various lengths of the polyhooks in the same cell.
(MOV)

Table S1 **FlgE peptides identified in isolated PFs**. Isolated PFs were run on SDS PAGE, excised, trypsin-digested and analyzed by MS as described. Those peptides identified with >95% confidence (conf) are shown, highlighted in green.
(XLSX)

Table S2 **FlgE peptides identified in isolated polyhooks**. Polyhooks were isolated from the *fliK* mutant and run on SDS PAGE, excised, trypsin-digested and analyzed by MS as described. Those peptides identified with >95% confidence (conf) are shown, highlighted in green.
(XLSX)
